# The new 222‐nm far ultraviolet‐C lowers bacterial contamination to endoscopists during esophagogastroduodenoscopy

**DOI:** 10.1002/deo2.292

**Published:** 2023-09-12

**Authors:** Yukari Fukutoku, Hidezumi Kikuchi, Kentaro Hoshi, Kouji Narita, Taka Asari, Kuniaki Miyazawa, Yohei Sawada, Shiro Hayamizu, Tetsuya Tatsuta, Shinji Oota, Keisuke Hasui, Hiroto Hiraga, Daisuke Chinda, Tatsuya Mikami, Phawinee Subsomwong, Krisana Asano, Kyosuke Yamane, Yoshimasa Ogawa, Masahiro Sasaki, Toru Koi, Hiroyuki Ohashi, Akio Nakane, Hirotake Sakuraba

**Affiliations:** ^1^ Department of Gastroenterology and Hematology Hirosaki University Graduate School of Medicine Aomori Japan; ^2^ Department of Community Medicine Hirosaki University Graduate School of Medicine Aomori Japan; ^3^ Department of Microbiology and Immunology Hirosaki University Graduate School of Medicine Aomori Japan; ^4^ Institute for Animal Experimentation Hirosaki University Graduate School of Medicine Aomori Japan; ^5^ Department of Preemptive Medicine Hirosaki University Graduate School of Medicine Aomori Japan; ^6^ Healthy Life Support Department, Marketing Division, Business Creation Division, Corporate Headquarters Ushio Inc. Tokyo Japan; ^7^ Department of Biopolymer and Health Science Hirosaki University Graduate School of Medicine Aomori Japan

**Keywords:** bacteria, endoscopy, incidence, infection control, ultraviolet rays

## Abstract

**Objectives:**

This study aimed to clarify the disinfectant efficacy of the 222‐nm far ultraviolet‐C (UV‐C) during esophagogastroduodenoscopy using bacterial cultures.

**Methods:**

The endoscopists performed esophagogastroduodenoscopy wearing a gown with a tryptic soy agar medium plate on their epigastric region and were divided into two groups: 222‐nm far UV‐C irradiation (UV group) and non‐UV irradiation (non‐UV group). As a control group, tryptic soy agar medium plates were placed about 110 cm above the floor. The incidence of bacterial contamination was determined by positive bacterial culture. The cultured bacteria were identified by 16S rRNA sequencing. Additionally, the actual UV exposure dose was measured using the UV‐indicator card which changed colors upon exposure to 222 nm far UV‐C.

**Results:**

The bacterial culture positivity in the UV group (5.03%) was significantly lower than that in the non‐UV group (25.76%), *p* < 0.0001. Most of the bacteria identified in the UV and non‐UV groups were normal constituents of the oral flora, including *Streptococcus salivarius* and *Staphylococci*. Conversely, pathogenic microbes were found in the control group. The actual exposure doses of 222‐nm far UV‐C at the endoscopists’ face, neck, and epigastric region were 2.09 ± 0.29, 5.89 ± 0.49, and 7.36 ± 0.58 mJ/cm^2^, respectively.

**Conclusions:**

The 222‐nm far UV‐C irradiation reduced bacterial contamination for endoscopists. It can be used with conventional physical coverings to provide more effective infection control.

## INTRODUCTION

Esophagogastroduodenoscopy (EGD) is a procedure that causes droplets and aerosols due to patients’ coughing or vomiting reflexes.^1^, ^2^, ^3^ Therefore, healthcare workers are at a high risk of contamination with microorganisms derived from patients’ secretions. Because of the coronavirus disease 2019 (COVID‐19) pandemic, this problem has become a major issue. Nowadays, various infection control methods are used in addition to wearing personal protective equipment during endoscopy. Previous studies have demonstrated that endoscopists are at high risk of contamination due to patients’ body fluids scattering,^4^ causing bacteria to spread on the endoscopist's face during an endoscopy.^3^ A case report has shown that bacterial exposure to the mucous membrane of the eyes can cause conjunctivitis.^5^

Currently, endoscopists perform endoscopies wearing personal protective equipment, which comprises a gown, gloves, mask, cap, and face shield. During the COVID‐19 pandemic, the American Society for Gastrointestinal Endoscopy recommended postponing endoscopies in non‐emergency cases, attachment and appropriate detachment of personal protective equipment, and screening for patients’ conditions and body temperatures before the procedures.^6^ In addition, the patient should wear a mask that is specifically cut to allow an endoscope to pass through, and an aerosol box should be used to cover the patient's head.^7^, ^8^, ^9^, ^10^ These conventional devices are considered physical coverings.

Ultraviolet (UV) light inactivates microbes by directly damaging their DNAs and RNAs.^11^, ^12^, ^13^, ^14^, ^15^ In particular, DNA is sensitive to 250₋260‐nm UV, and conventional 254‐nm UV has been shown to be effective for the inactivation of bacteria. However, it also affects the DNA of human cells, causing adverse events such as skin cancer and cataracts.^16^, ^17^

Recently, 222‐nm far UV‐C has gained attention as a decontamination method.^18^ The light is absorbed by the stratum corneum of the skin without reaching the DNA of living cells because the absorption coefficient of protein is high for 222‐nm far UV‐C. To our knowledge, no data have yet been reported on the side effects of 222‐nm far UV‐C on the stratum corneum of the skin. Meanwhile, 222‐nm far UV‐C easily reaches microbial DNA and RNA because a microbe is extremely small. In other words, 222‐nm far UV‐C can inactivate bacteria and viruses as efficiently as 254‐nm UV‐C while being conclusively safe for humans.^19^, ^20^, ^21^, ^22^, ^23^, ^24^, ^25^, ^26^, ^27^, ^28^, ^29^, ^30^ Therefore, 222‐nm far UV‐C may constitute a new infection control method in a human‐occupied area.^31^

To our knowledge, no study has reported infection control using UV irradiation in hospital settings during endoscopy. Herein, we investigated the bacterial contamination of endoscopists during EGD under 222‐nm far UV‐C. In addition, the exposed irradiation dose of 222 nm was calculated using UV‐indicator cards.

## METHODS

### Endoscopy procedure

This single‐center, retrospective study included and assessed endoscopy procedures performed at the Division of Endoscopy, Hirosaki University Hospital, between March 15 and October 28, 2021. This study consisted of a survey of bacterial contamination in the endoscopy room and a retrospective analysis of clinical information. The study was approved by the ethics committee of Hirosaki University Graduate School of Medicine (No. 2022–076) and conducted in accordance with the Declaration of Helsinki. Each patient was informed that the endoscopy room would use 222‐nm far UV‐C for infection control, and only after obtaining their consent to participate, did the patient undergo an EGD. We retrospectively analyzed the clinical information with approval from the ethics committee.

**FIGURE 1:**
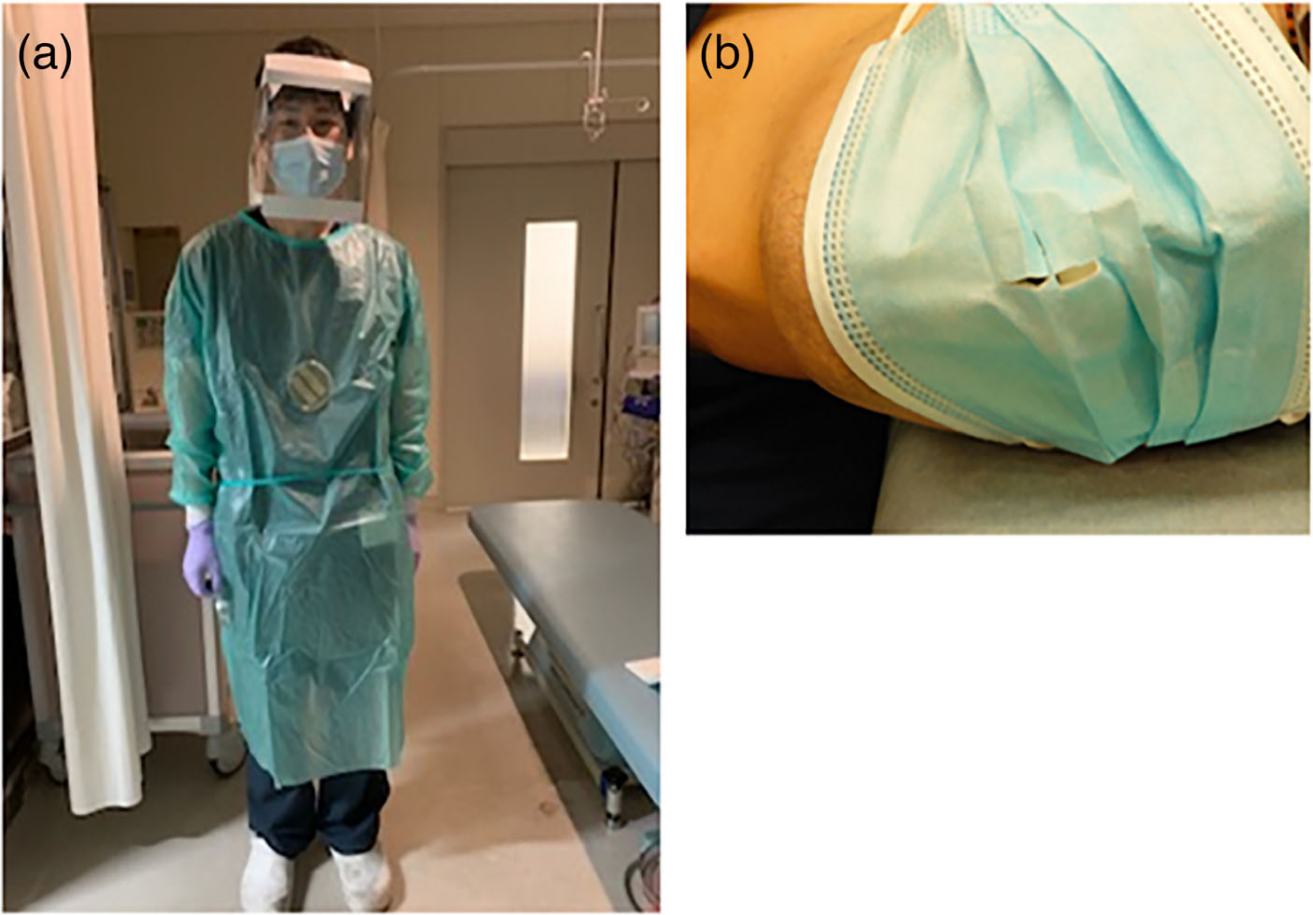
Endoscopy procedure. (a) The endoscopists performed esophagogastroduodenoscopy while wearing a gown with a tryptic soy agar medium plate attached to their epigastric region. The tryptic soy agar medium plate was approximately 110 cm above the floor. (b): The patients wore a mask cut in the center to allow the endoscope to pass during esophagogastroduodenoscopy.

The surveyed EGD were performed on Wednesdays or Fridays in an endoscopy room (No. 1 or 2 out of No. 1–4) which was randomly allocated by a medical clerk. Endoscopy rooms No. 1 and No. 2 were separated by a curtain. The endoscopists performed EGD wearing a gown with a petri dish containing tryptic soy agar medium (TSA) attached to their epigastric region at a height of about 110 cm above the floor (Figure 1a). The EGD was divided into two groups according to the endoscopy room used: 222‐nm far UV‐C irradiation group (UV group) in endoscopy room No. 2 and the non‐UV irradiation group (non‐UV group) in endoscopy room No. 1.

This study categorized EGD into two types: diagnostic endoscopy (DE) and therapeutic endoscopy (TE). DE included standard diagnostic procedures, such as magnifying endoscopy, chromoendoscopy, digital image enhanced endoscopy, forceps biopsy, pre‐operation marking, and endoscopic ultrasonography. TE included endoscopic submucosal dissection (ESD), hemostasis, and foreign matter removal. Generally, TE needs a longer examination time than DE. To accurately evaluate the effect of 222‐nm far UV‐C regardless of irradiation time, each TE procedure was surveyed for up to 10 min.

In addition, as a control group (C group), environmental bacteria in the endoscopy rooms were evaluated by placing a TSA plate at a height of approximately 110 cm above the floor for 10 min during non‐endoscopic procedures. This height was used because the epigastric regions of endoscopists were almost 110 cm high. The bacteria were collected from the same height in the UV, non‐UV, and C groups.

### Clinical information

To compare the clinical information between the UV and non‐UV groups, patient information, endoscopists’ qualifications, and sedation used during endoscopy were retrospectively collected from medical reports. Endoscopists were classified as specialists, board‐certified fellows of the Japan Gastroenterological Endoscopy Society, and non‐specialists. Sedatives were used during endoscopy when necessary and included benzodiazepines, pethidine, and dexmedetomidine hydrochloride. In this study, all patients wore a mask that was cut in the center to allow endoscope passage (Figure 1b).

### Settings of 222‐nm far UV‐C light lamp

**FIGURE 2:**
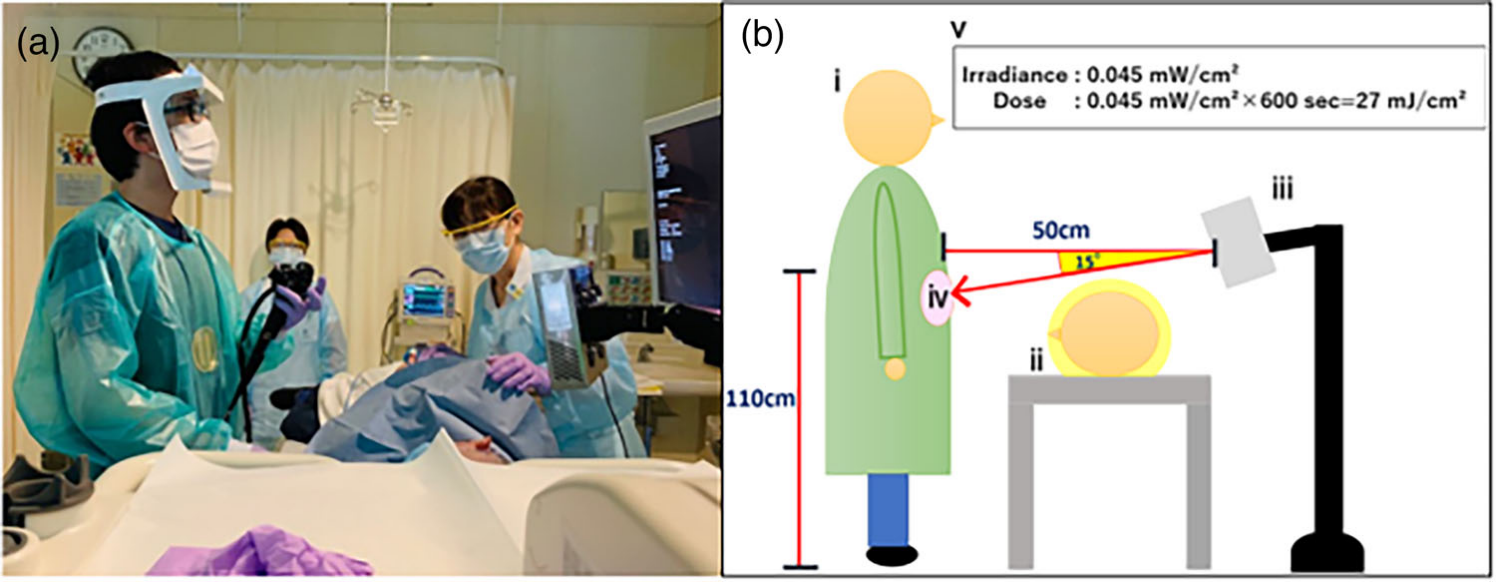
Setting of the 222‐nm far UV‐C light lamp. (a) Care222® is used as shown in the picture. (b) i: Endoscopist. ii: Patient reclining on their left arm on the inspection table. iii: A Care222® device placed behind the patient and mounted to a pole at a height of 120 cm above the floor at a 15‐degree angle below the horizontal plane. iv: Endoscopists performed esophagogastroduodenoscopy while wearing the gown with a tryptic soy agar medium plate attached to their epigastric region. The tryptic soy agar medium plate was approximately 110 cm above the floor. v: The exposure dose reaching the endoscopist's epigastric region was 27 mJ/cm^2^ after a 10‐min continuous irradiation at 45 μW/cm^2^.

The Care222® (Ushio Inc.) 222‐nm far UV‐C lamp, which irradiates continuously at an irradiance of 45 μW/cm^2^, was used in endoscopy room No. 2 (Figure 2a). A Care222® device was mounted to a pole at a 15‐degree angle below the horizontal plane and placed behind the patient (Figure 2b). The distance from the endoscopist was 50 cm. The endoscopist's epigastric region was irradiated for 10 min to achieve an exposure dose of 27 mJ/cm^2^. When the examination time was within 10 min, UV irradiation was stopped at the same time when the endoscopy was completed. The exposure dose reached the level of sufficient anti‐bacterial performance, which was defined as bacterial 3‐log reduction.^13^

### Bacterial culture and bacterial species identification

To prepare the TSA, 12.0 g tryptic soy broth powder and 6.0 g agar powder were added to 400 mL distilled water and autoclaved. The solution was then divided into 20 petri dishes and allowed to cool and solidify. The endoscopists attached a TSA plate to their epigastric region. After EGD, the collected TSA plates were incubated at 37°C for 24 h, and the colony‐forming positive ratio and colony‐forming unit (CFU) were evaluated. The microorganisms cultured from July 5 to September 9, 2021, were identified using 16S rRNA sequencing methods (Macrogen JAPAN).

### Measurement of the 222‐nm far UV‐C exposure dose to the endoscopists

**FIGURE 3:**
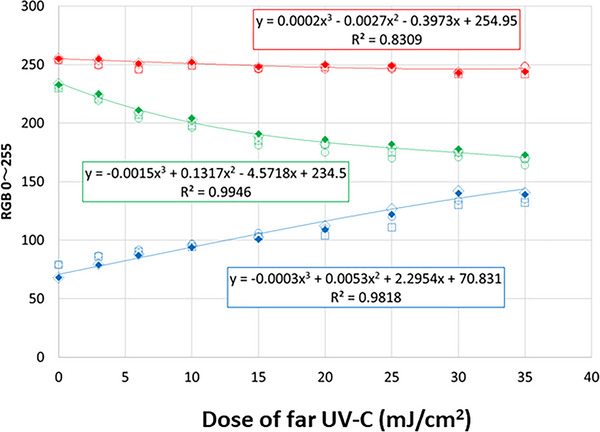
Standard curves. The three standard curves are shown in red, green, and blue. R^2^ = correlation coefficient.

DOSE222™ (Ushio America Inc.) is a UV‐indicator card that changes colors by irradiation with 222‐nm far UV‐C. For preliminary experiments, 222‐nm far UV‐C was irradiated by Care222® to the UV‐indicator cards at an irradiance of 45 μW/cm^2^ and in exposure doses of 0, 3, 6, 10, 20, 30, and 35 mJ/cm^2^. The exposed areas on the UV‐indicator cards were scanned using a digital IM C5500 full‐color composition machine (RICOH) to create still‐image data, formatted as Joint Photographic Experts Group files, at a resolution of 400 dots per inch. The three primary colors (red [R], green [G], and blue [B]) were extracted from a 20 × 20‐pixel area of the UV‐indicator card images using the Image J.^32^ From these preliminary experiments, the standard curves of the cubic equation were constructed for all three primary colors (Figure 3).

**FIGURE 4:**
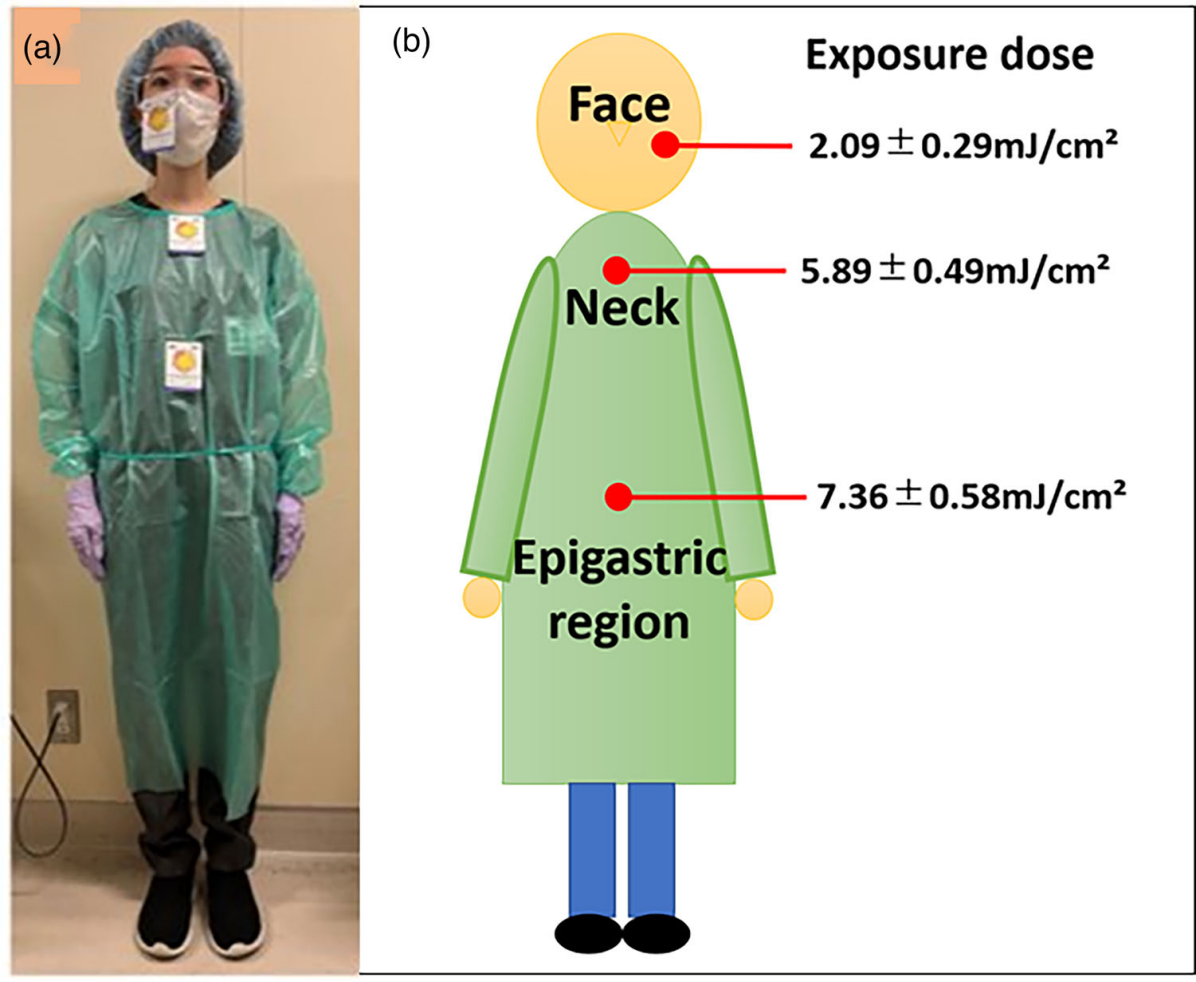
Exposure doses. (a) The UV‐indicator cards were attached to three body parts₋face, neck, and epigastric region of the endoscopists during esophagogastroduodenoscopy (EGD). (b) The exposure dose at the face, neck, and epigastric region were 2.09 ± 0.29, 5.89 ± 0.49, and 7.36 ± 0.58 mJ/cm^2^, respectively.

To measure the exposure dose of 222‐nm far UV‐C, the endoscopists attached the UV‐indicator cards on three body parts₋face, neck, and epigastric region during EGD performed between July 29 and October 14, 2022 (Figure 4a). After the EGD, the UV indicator cards were immediately collected and scanned. The acquired image data were separated by R, G, and B primary color levels, and the actual exposure doses were measured using the standard curves.

### Statistical analyses

All statistical analyses were performed using chi‐square tests, Student's t‐test, and Mann‐Whitney U test with EZR (Saitama Medical Center, Jichi Medical University, Saitama, Japan)^33^; this is a graphical user interface for R (The R Foundation for Statistical Computing), which is a modified version of R commander designed to compute frequently used statistical functions in biostatistics. The CFU and the exposure dose were presented as average ± standard error. The threshold for significance was set at *p* < 0.05.

Ushio Inc. handled the Care222® setting and co‐conducted UV‐C dose measurements; however, Ushio Inc. did not analyze the data regarding the effects of UV‐C.

## RESULTS

### Clinical information

The clinical information regarding the surveyed EGD is summarized (Table 1). A total of 271 procedures were performed in the UV (*n =* 139) and non‐UV (*n =* 132) groups. The UV group had 130 DE (diagnostic EGD; *n =* 128, pre‐operation marking; *n =* 2) and nine TE (ESD; *n =* 7, hemostasis; *n =* 2). The non‐UV group had 128 DE (diagnostic EGD; *n =* 124, pre‐operation marking; *n =* 2, endoscopic ultrasonography; *n =* 1, endoscopic ultrasonography fine‐needle aspiration; *n =* 1) and four TE (hemostasis; *n =* 3, foreign matter removal; *n =* 1). The clinical information was not significantly different between the UV and non‐UV groups. The C group involved 126 TSA plates.

**TABLE 1:** Clinical information.

Clinical information	UV (*n =* 139)	Non‐UV (*n =* 132)	*p*‐value
Patient's median age, years (range)	70 (31–87)	70 (19–85)	0.497
Patient's sex, male/female	98/41	79/53	0.086
Types of endoscopies, DE/TE	130/9	128/4	0.298
Endoscopists, specialist/non‐specialist	69/70	54/78	0.187
Sedation during endoscopy, without/with	118/21	117/15	0.466

Abbreviations: UV, ultraviolet; DE, diagnostic endoscopy; TE, therapeutic endoscopy.

### Bacterial contamination incidence

**FIGURE 5:**
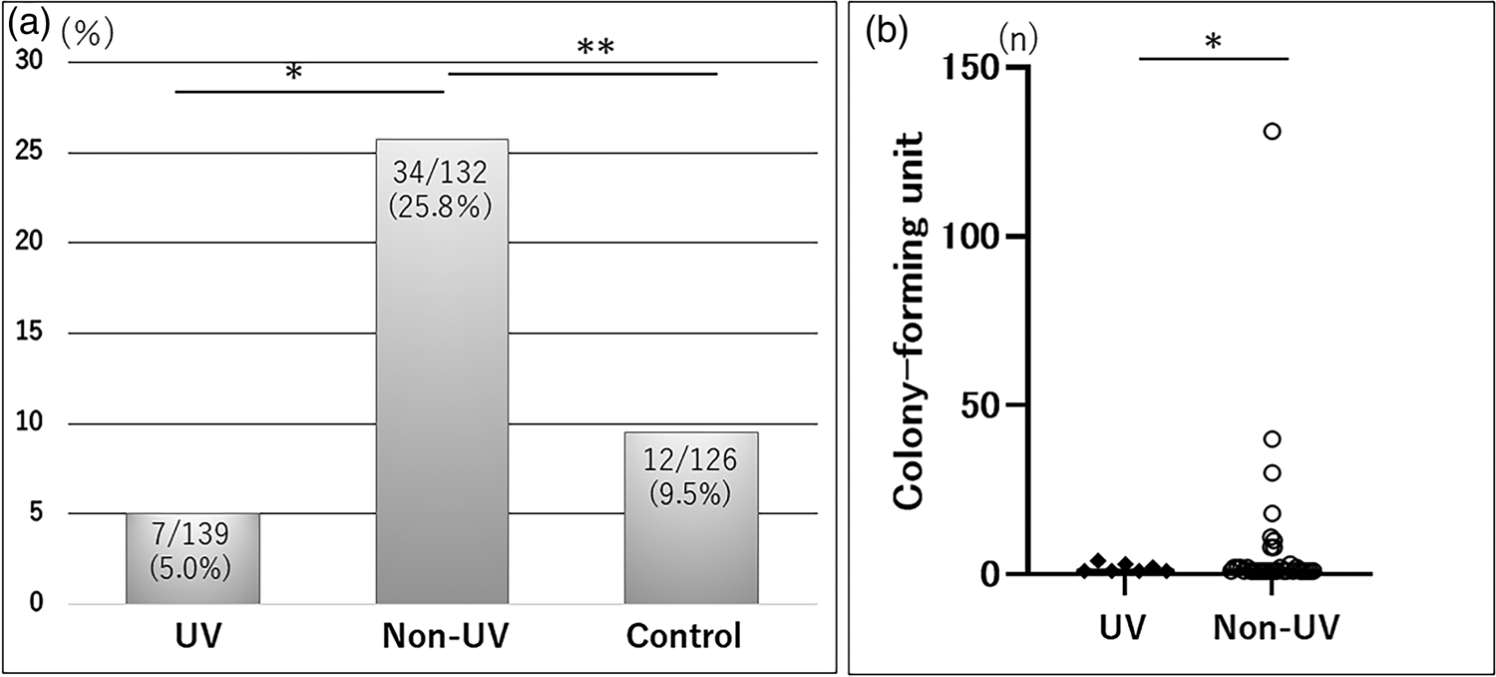
Bacterial contamination analysis. (a) Bacterial culture positivity; *p = 0.000004461, **p = 0.00066. (b) Colony‐forming units in the ultraviolet (UV) and the non‐UV groups were 0.09 ± 0.04 and 2.20 ± 1.15, respectively; *p = 0.000002.

The bacterial culture positivity was significantly lower in the UV group than in the non‐UV group (5.03% [7/139] vs. 25.76% [34/132], *p* < 0.0001; Figure 5a). The bacterial culture positivity in the C group was 9.52% (12/126) which was significantly lower than that in the non‐UV group (*p* < 0.001). There was no significant difference in the bacterial culture positivity between the UV and C groups. Furthermore, the CFU was significantly lower in the UV group (0.09 ± 0.04) than in the non‐UV group (2.20 ± 1.54, *p* < 0.0001; Figure 5b).

### Identification of bacterial species

Bacterial cultures were positive in 21 cases (four in the UV group, 13 in the non‐UV group, and four in the C group). In the non‐UV group, polymerase chain reaction could not identify the bacteria species in four cases, whereas two cases had two types of bacteria identified in each case. Overall, 19 bacterial species were identified (Table 2).

**TABLE 2:** Identification of bacterial species.

Bacterial species	UV	Non‐UV	Control	Total (*n*)
*Streptococcus salivarius*	1	4	0	5
*Bacillus subtilis*	1	1	1	3
*Staphylococcus hominis*	1	0	1	2
*Staphylococcus epidermidis*	0	2	0	2
*Neisseria* sp.	1	0	0	1
*Neisseria subflava*	0	1	0	1
*Hafnia paralvei*	0	1	0	1
*Staphylococcus aureus*	0	1	0	1
*Staphylococcus saprophyticus*	0	1	0	1
*Micrococcus* sp.	0	0	1	1
*Enhydrobacter aerosaccus*	0	0	1	1

Abbreviation: UV, ultraviolet.

In the samples, *Streptococcus salivarius* (*n =* 5) and *Staphylococci* (*n =* 6) were the most commonly found bacteria identified by 16S rRNA gene sequencing. The identified *Staphylococci* included *Staphylococcus epidermidis* (*n =* 2), *Staphylococcus hominis* (*n =* 2), *Staphylococcus aureus* (*n =* 1), and *Staphylococcus saprophyticus* (*n =* 1).

### Exposure dose of 222‐nm far UV‐C irradiation

From the preliminary experiments, a standard curve was obtained for each of the three primary colors. Among them, G produced the highest quality color level, based on the standard curves. Therefore, the exposure doses of 222‐nm far UV‐C were measured using the G level of the UV‐indicator cards in this study.

The calculated exposure doses of 222‐nm far UV‐C to the endoscopists in 22 EGD procedures are shown in Figure 4b. The exposure doses to the face, neck, and epigastric region were 2.09 ± 0.29, 5.89 ± 0.49, and 7.36 ± 0.58 mJ/cm^2^, respectively.

## DISCUSSION

To our knowledge, this is the first study demonstrating the antibacterial effect of 222‐nm far UV‐C in a hospital setting. Herein, 222‐nm far UV‐C significantly reduced bacterial contamination during EGD from 25.76 to 5.03%. Previously, we had reported bacterial contamination to an endoscopist's face during EGD to be 11.5%.^34^ This study showed that bacterial contamination to the endoscopist's epigastric region during EGD was 25.76%. The high risk of bacterial contamination to endoscopists was significantly decreased by 222 nm far UV‐C. Most of the antibacterial effects of 222‐nm far UV‐C have been shown in animal models or bacterial studies. The present study demonstrated similar antibacterial effects of 222‐nm far UV‐C in a hospital setting.

There was a significant difference in CFU between the UV and non‐UV groups, suggesting that 222‐nm far UV‐C reduced the number of contaminating bacteria even if bacterial exposure occurred. In addition, high CFU values (>10) were observed in six cases in the non‐UV group. In five out of the six cases, endoscopists performed examinations with working channel techniques. This indicates that splattering from the working channel can lead to contamination with high bacteria counts.

However, in the UV group, not all bacterial contamination disappeared. One of the reasons why a few bacteria remained may be that the UV rays were irradiated in a straight line; therefore, the shadows cast by the endoscopist's postures might have blocked the UV rays and influenced their effect. Another reason is that the contamination might have occurred just before the end of the EGD. EGD often causes patients to belch and cough during endoscope removal, which can be a source of bacterial scattering. The UV‐C irradiation cannot reach the sufficient dosage required for antibacterial effect if bacterial contamination occurs at the end of the EGD. Therefore, the combination of functional antimicrobial methods, such as 222‐nm far UV‐C, and physical methods, such as conventional coverings, would be strongly effective as an infection control method.

Most of the identified bacterial species in the UV and non‐UV groups were normal constituents of the oral flora. Previous reports have shown that *Streptococcus salivarius*, *Staphylococcus epidermidis*, and *Bacillus subtilis* are detectable in the human mouth.^35^ Therefore, some of the identified bacteria in these groups could have been scattered from the patient's mouth, even though the patient was wearing a mask. Contrarily, the bacterial species identified in the C group included microbes often found in environments such as soil, water, and air. The bacterial culture positivity of the UV group tended to be lower than that of the C group, suggesting that 222‐nm far UV‐C could reduce the bacterial contamination to healthcare workers from both the patients and environments.

According to the initial setting of Care222©, the exposure dose to the endoscopists' epigastric region was expected to be 27 mJ/cm^2^ after 10 min of continuous irradiation; however, the exposure dose was lower, probably because of the movements or postures of the endoscopist. Thus, this study also showed that 222‐nm far UV‐C reduced bacterial contamination to the endoscopists even at a lower exposure dose.

As for 222‐nm far UV‐C, although the safety for living cells is confirmed, its daily exposure dose limit is the same as that of conventional UV. The upper limits of conventional UV exposure dose set by the International Electrotechnical Commission are as follows: the threshold limit value (TLV) of 254‐nm UV‐C is 6 mJ/cm2, and the TLV of 222‐nm far UV‐C is 22 mJ/cm^2^. However, in 2022, the American Conference of Governmental Industrial Hygienists recommended a threshold of 160 mJ/cm^2^ for 222‐nm far UV‐C when naked eyes and skin are exposed, and of a threshold 479 mJ/cm^2^ if the eyes were protected.^36^ In our study, the actual exposure doses were within these two parameters; therefore, the risk of overexposure to 222‐nm far UV‐C for endoscopists was small.

Our study also showed the weakness of 222‐nm far UV‐C. First, UV rays are radiated linearly; therefore, the irradiated area is limited. Second, bacterial spreading just before the end of the procedure may not be prevented because of insufficient irradiation time.

In several previous reports, a lower exposure dose of 222 nm far UV‐C was required to eliminate viruses compared with bacteria.^22^, ^37^, ^38^ Since the COVID‐19 pandemic, viral infections from patients to healthcare workers have become a major problem. The 222‐nm far UV‐C is therefore expected to reduce viral infection risks during endoscopies.

The limitations of this study include the single‐center nature and a small number of cases. Moreover, we could not detect all microorganisms. As TSA only allows for bacterial growth, we could not detect microorganisms such as fungi or viruses. In addition, it is difficult to determine whether the cultured bacteria were derived from patient fluids. A small possibility of contamination from the adjacent room remained because the two rooms were only separated by a curtain. Moreover, because UV light is invisible, ascertaining whether it reached the targeted area was not feasible. Our results should be interpreted while considering factors such as the endoscopist's height and the patient's position. Furthermore, future studies may be needed to reveal the side effects of 222‐nm far UV‐C on the stratum corneum of the skin.

In conclusion, our study demonstrates that 222‐nm far UV‐C has a disinfecting effect against bacterial contamination during EGD in a hospital setting. Therefore, the combination of 222‐nm far UV‐C along with conventional physical coverings is expected to be an effective infection control measure. In the future, additional data regarding the effects of 222‐nm far UV‐C in clinical practice are expected.

## CONFLICT OF INTEREST STATEMENT

Kyosuke Yamane, Yoshimasa Ogawa, Masahiro Sasaki, Toru Koi, and Hiroyuki Ohashi are full‐time employees of Ushio Inc.
